# Green’s Pathology

**Published:** 1906-02

**Authors:** 


GREEN'S PATHOLOGY. Tenth edition. A Text-book of Pathology and Pathological Anatomy. By T. Henry Green, M.D., F. R. C. P., consulting Physician to Charing Cross Hospital, London. New (10th) edition. Thoroughly revised by W. Cecil Bosanquet, A.M., M.D., F. R. C. P., Assistant Physician to Charing Cross Hospital. Octavo, 606 pages, 348 engravings and a colored plate. Cloth, $2.75, net. Lea Brothers & Co., Publishers, Philadelphia and New York, 1905.

This book furnishes a clear, adequate presentation of modern pathology, and the frequent editions that have been required to supply the demand for such a work made it possible for it to be kept well abreast the times. The eminence of its author and revisors is a sufficient guarantee of its merits and reliability.

There are few branches of medicine or surgery that have undergone such rapid transformations as pathology and it is only by frequent revisions that a book can be kept at all up-to-date. In the above work all the newer discoveries have been added and especially notable are those in animal parasites and immunity. Erlich's theory is briefly but concisely discussed.

As a whole this book may be taken as a fair index of the well-established facts in pathology.

